# Successful Treatment of Painful Synchondrosis of Bipartite Patella after Direct Trauma by Operative Fixation: A Series of Six Cases

**DOI:** 10.2174/1874325001711010390

**Published:** 2017-05-17

**Authors:** Sarkhell Radha, Michael Shenouda, Sujith Konan, Jonathon Lavelle, Samuel Church

**Affiliations:** Department of Trauma & Orthopaedics, Chelsea & Westminster Hospital, London, UK

**Keywords:** Anterior knee pain, Bipartite patella, Synchondrosis rupture, Operative fixation, Patellofemoral joint, Trauma

## Abstract

**Introduction:**

The patella is the largest sesamoid bone in the body and may have one (77%) or multiple (23%) ossification centres. Patellar and patellofemoral joint abnormalities are a common cause of anterior knee pain but symptomatic bipartite patella is an uncommon problem.

**Case Series:**

We report a series of six cases of painful synchondrosis in bipartite patellae, all in keen athletes following a direct blow to the anterior aspect of the knee. A complete rupture of the synchondrosis with evidence of retropatellar chondral separation was seen on MRI scan in all cases. Successful surgical fixation was undertaken with complete resolution of symptoms in all patients at an average of three months post-operatively.

**Conclusion:**

Painful synchondrosis of a bipartite patella in young and active individuals following direct trauma is a relatively rare cause of anterior knee pain, but may be associated with significant morbidity. In cases refractory to non-operative management, successful symptomatic treatment can be achieved by operative fixation.

## INTRODUCTION

Bipartite patella is commonly asymptomatic and often an incidental finding on radiological imaging. The patella remains bipartite when secondary ossification centres stop fusing to form a single patella, and may be bilateral in up to 50% of cases [1]. Symptomatic bipartite patella usually occurs in adolescent males or young athletes and may be a cause of anterior knee pain [2].

Particularly in active individuals, the patellar tendon and quadriceps muscles produce extensive forces on the patella. This, combined with vertical patellar restraining forces from the medial patellofemoral ligament, results in constant distraction and friction along the patellar synchondrosis [3]. This can lead to inflammation and an MRI finding of bone marrow oedema, with resultant anterior knee pain. The condition “painful synchondrosis” is a diagnosis of exclusion and should be considered in patients with anterior knee pain and otherwise normal findings on examination and plain radiographs.

We present a series of six patients with symptomatic anterior knee pain secondary to painful synchondrosis of a bipartite patella. All patients gave a history of a direct blow to the anterior aspect of the knee and had failed to respond to conservative treatment. The MRI scans demonstrated disruption of the entire synchondrosis and the underlying articular cartilage. All patients were treated successfully with fusion and achieved complete resolution of symptoms at 3 months follow-up. One patient required removal of screws due to irritation of the vastus medialis muscle insertion at 12 months post-op.

## CASE SERIES

Between 2006 and 2011, six patients were referred to our specialist knee unit with undiagnosed anterior knee pain. Two of these patients were keen football players, and four were keen runners. All were male patients, with an age range of 19-26 years old at presentation. All patients gave a history of a direct blow to the anterior aspect of the knee while playing sport or following a fall whilst running. They reported no prior history of knee pain and were all otherwise fit and well.

**Fig. (1):**
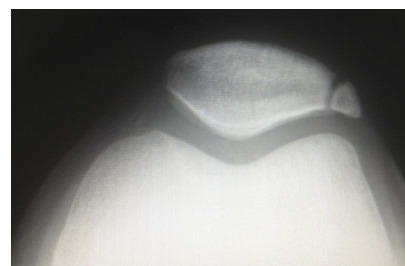
Initial radiograph in a patient with anterior knee pain following fall onto left knee, showing bipartite patella with no bony abnormalities.

**Fig. (2):**
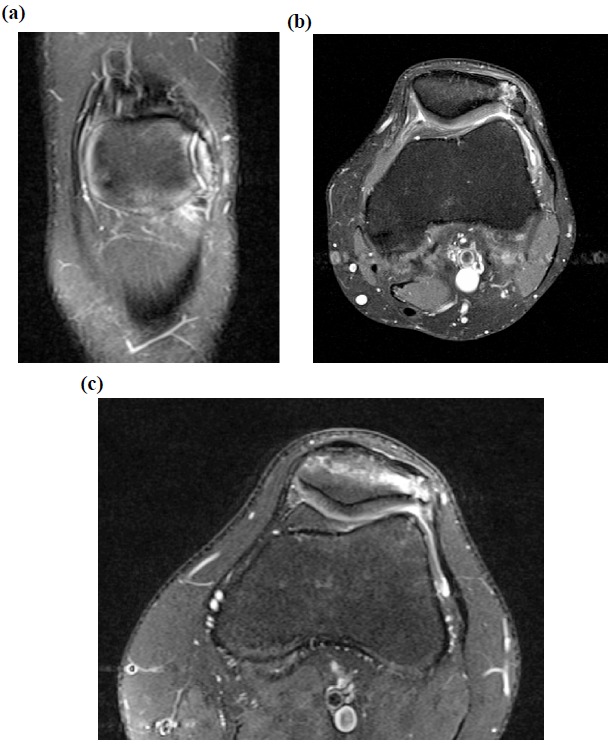
Pre-operative MRI images showing bipartite patella with disruption of the synchondrosis and a fissure in the underlying articular cartilage.

Physical examination was unremarkable apart from localised tenderness over the patellar synchondrosis. Plain radiography in all cases confirmed a bipartite patella with no evidence of obvious other bony pathology (Fig. **1**). All cases were of unilateral bipartite patella. The patients were investigated with MRI scans which revealed a rupture of the synchondrosis and adjacent bone marrow oedema with retropatellar chondral separation (Figs. **2a**, **b**, **c**).

In all 6 patients, the pain remained refractory to non-surgical management including rest, physiotherapy, ultrasound-guided local anaesthetic and steroid injections by an experienced musculoskeletal radiologist (40mmol Depomedrol, used in 2 cases, as previously recommended by Weaver, 1977 [4] and Ogata, 1994 [5]) and non-steroidal anti-inflammatory drugs. Non-surgical options were trialled for a mean period of four months (range 3-12 months).

**Fig. (3):**
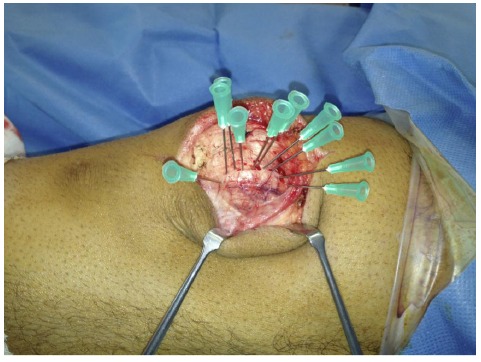
Intra-operative photograph showing multiple green needles being used to identify the plane of the synchondrosis.

**Fig. (4):**
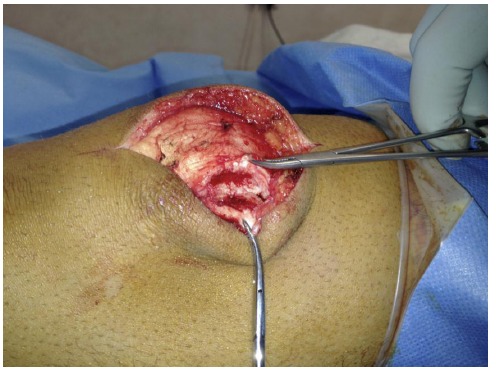
Intra-operative photograph showing the retinaculum overlying the synchondrosis being divided and opened like a book, to allow curettage and decortication.

**Fig. (5):**
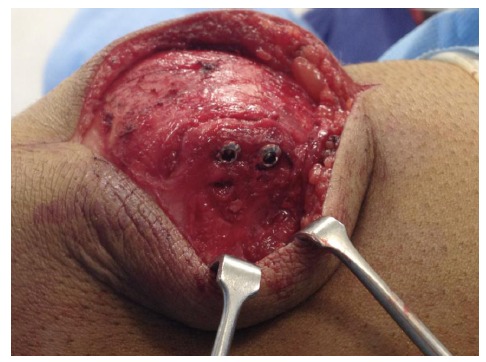
Intra-operative photograph showing operative fixation using two cortical screws.

The patients were therefore counselled and consented for operative fixation, and all were treated with open reduction and internal fixation using 3.5 mm cortical screws. The procedures were performed via a midline longitudinal incision. Multiple green needles were used to identify the plane of the synchondrosis (Fig. **3**). The retinaculum overlying the synchondrosis was divided, allowing it to be opened like a book (Fig. **4**) and exposing the synchondrosis. This was debrided back to cancellous bone on both sides of the synchondrosis. The bipartite fragment was then reduced to the patella using a fracture reduction clamp. Fixation was achieved with fully threaded cortical lag screws (Fig. **5**); no bone graft was required. There were no intra-operative complications.

Post-operatively, mobilisation was allowed using a range-of-movement brace from 0-30 degrees of flexion for 2 weeks, increasing to 90 degrees of flexion by 6 weeks. The brace was then removed and patients were encouraged to continue lower limb strengthening exercises. Regular manual patellar glides were encouraged throughout this period.

**Fig. (6):**
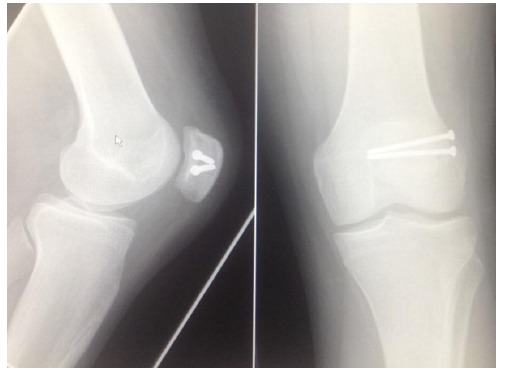
Post-operative radiographs taken at 6 weeks.

Clinical and radiological union was achieved at 6 weeks follow-up (Fig. **6**). All patients had complete symptomatic relief at 3 months. Clinically, full knee function was restored in all cases, with return to previous level of activity by 6 months, and patients were discharged. One patient required proximal screw removal due to hardware irritation at 12 months post-operatively.

## DISCUSSION

Bipartite patella is a common incidental finding; it occurs in 2-3% of the population and can be bilateral in 50% of cases [1, 6]. A bipartite patella results from an accessory ossification centre that persists as a separate fragment, attaching to the patella through a fibrocartilaginous joint or interface. This accessory ossification centre appears at 8-12 years of age [1]. A bipartite patella is usually asymptomatic, but the literature suggests that approximately 2% of cases may present as anterior knee pain [2, 4]. Patients are usually young men below the age of 20 who are actively involved in sporting activities [4, 7, 8], although rarer cases of painful bipartite patella have also been reported in older age groups following strenuous sports or separation secondary to direct trauma [5, 9]. The onset of anterior knee pain can be acute following direct trauma, or insidious as a result of indirect injury over a prolonged period of time with sporting activities such as cycling and running [10].

The fibrocartilaginous zone between the patella and the bipartite fragment is known as the synchondrosis. The interposed tissue between the two cartilaginous surfaces can be fibrous [11], fibrocartilaginous [11] or hyaline cartilage [7]. Canizares and Selesnick [12] reported reactive and degenerative changes within this fibrocartilaginous structure leading to anterior knee pain. The precise mechanism of such degenerative changes is not understood and interference with diffusion of nutritive fluid through this fibrocartilaginous structure has been implicated [3].

Plain radiographic findings are often inconclusive although they may reveal widening of the synchondrosis on the skyline view [13]. MRI scanning can provide more information by showing bone marrow oedema at the interface between the bipartite fragment and the patella, as described in our case series. This finding has never been described in the asymptomatic bipartite patella [13]. Furthermore, the disruption of the retropatellar articular cartilage seen on MRI in our case series has not yet been described in the literature. Our experience shows that MRI scanning provides invaluable information and should be considered in patients presenting with anterior knee pain localising to a bipartite patella.

There is controversy with regards to the causative mechanism of painful bipartite patella. As this condition most commonly occurs in patients who take part in repetitive physical activities such as running, it has been suggested that repetitive pivoting stress forces across the knee during these activities may cause lateral stress across the patella and distraction of the interface between the two bones [3]. Increased movement across abnormal synchondrosis has also been implicated in causing pain as it results in a thickened and traumatised vastus lateralis insertion over a period of time [5]. Cases of longitudinal patellar fracture in this area have also been described; it has been suggested that an intact medial patellofemoral ligament causes lateral stress in the patella which creates longitudinal fractures [14].

There is also controversy in the management of this condition. Conservative treatment options such as rest, modification of sporting activities, quadriceps exercises and steroid injections have been used as first-line treatments with variable results and high recurrence rates [2]. Excision of the small bipartite fragment, lateral retinacular release and detachment of the vastus lateralis insertion have also been reported to provide pain relief [1]. These procedures may cause abnormal patellofemoral biomechanics however, and the results are unpredictable at best.

Although several cases of painful bipartite patella have been managed conservatively in our unit with good results, all the cases reported in this series did not respond to such treatments. Interestingly, all these cases had a clear history of a direct blow to the anterior knee with evidence of disruption of the synchondrosis and fissuring of the retropatellar articular cartilage on MRI scan. The pathology here, we believe, is an unstable synchondrosis causing pain, as opposed to patients with an intact but painful synchondrosis, in whom conservative treatment may be more successful. We believe conservative management of cases with clear disruption of the synchondrosis, as evidenced by the MRI findings described, may be associated with limited improvement especially in active individuals. Operative fusion as described in our cases has proved successful in achieving long-term symptomatic relief and allowing these active patients to return to their respective level of sporting activity.

We therefore recommend that MRI scanning can contribute significantly to careful patient selection for operative fixation, which can allow sporting individuals to achieve full symptomatic relief, with return to premorbid activity levels and good long-term outcomes without any negative effects on patellofemoral biomechanics and stability.

The present study has limitations. These include the relatively small number of cases, a lack of comparative control group with alternative treatments, for example fragment excision, and a relatively limited period of follow-up. Further research, ideally in the form of randomised controlled trials, including documented knee scores and with longer term follow-up, is required to define the optimal treatment for this difficult condition.

## CONCLUSION

Painful patellar synchondrosis is a distinct clinical entity and may occur following direct trauma, resulting in decreased activity levels in active individuals. In carefully selected patients with a clear history of traumatic event, persistent symptoms despite appropriate conservative management, and with MRI evidence of complete disruption of the synchondrosis, operative fusion of the synchondrosis provides effective symptomatic relief and return to premorbid activity levels.
